# Supplementary material for: The interactome of intact mitochondria by cross-linking mass spectrometry provides evidence for coexisting respiratory supercomplexes
Source: Mol Cell Proteomics. 2017 Dec 8;17(2):216–32. doi: 10.1074/mcp.RA117.000470 (PMC5795388; doi:10.1074/mcp.RA117.000470)
Supplement: Supplemental Data [file supp_RA117.000470_133922_0_supp_23978_fzffwf.zip › spectra_annotation/mito_spectra_annotation/290-1-1-1-9-1.pdf]

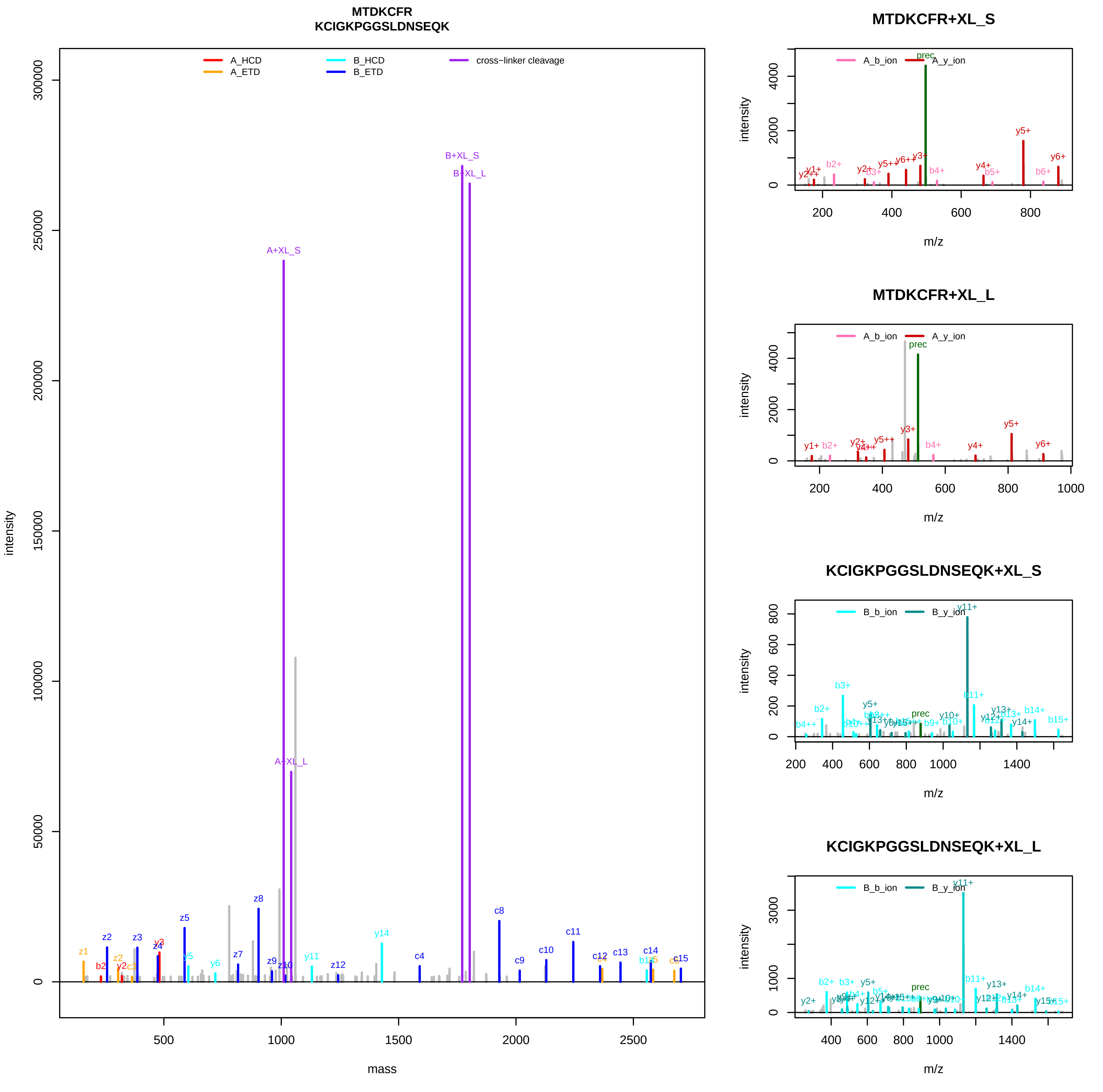
MTDKCFR
KCIGKPGGSLDNSEQK
A_HCD
A_ETD
B_HCD
B_ETD
cross−linker cleavage
B+XL_S
B+XL_L
A+XL_S
A+XL_L
z1
b2
z2
y2
c3
z3
z4
y3
z5
y5
y6
z7
z8
z9
z10
y11
z12
y14
c4
c8
c9
c10
c11
c12
c4
c13
c14
b15
c5
c6
c15
intensity
mass
MTDKCFR+XL_S
A_b_ion
A_y_ion
prec
y2++
y1+
b2+
y2+
b3+
y5++
y6++
y3+
b4+
y4+
b5+
y5+
b6+
y6+
m/z
MTDKCFR+XL_L
y1+
b2+
y2+
y4++
y5++
y3+
b4+
y4+
y5+
y6+
KCIGKPGGSLDNSEQK+XL_S
B_b_ion
B_y_ion
y11+
b3+
b2+
b4++
y5+
b11+
y13+
b14+
b15+
y10+
y12+
y14+
KCIGKPGGSLDNSEQK+XL_L
y2+
b2+
b3+
y5+
b5+
b11+
y13+
b14+
y14+
y15+
